# CD4+ and B Lymphocyte Expression Quantitative Traits at Rheumatoid Arthritis Risk Loci in Patients With Untreated Early Arthritis: Implications for Causal Gene Identification

**DOI:** 10.1002/art.40393

**Published:** 2018-01-30

**Authors:** Nishanthi Thalayasingam, Nisha Nair, Andrew J. Skelton, Jonathan Massey, Amy E. Anderson, Alexander D. Clark, Julie Diboll, Dennis W. Lendrem, Louise N. Reynard, Heather J. Cordell, Stephen Eyre, John D. Isaacs, Anne Barton, Arthur G. Pratt

**Affiliations:** ^1^ NIHR Newcastle Biomedical Research Centre Newcastle upon Tyne Hospitals NHS Foundation Trust, and Newcastle University Newcastle upon Tyne UK; ^2^ Arthritis Research UK Centre for Genetics and Genomics Centre for Musculoskeletal Research Institute of Inflammation and Repair University of Manchester and NIHR Manchester Musculoskeletal Biomedical Research Unit Central Manchester NHS Foundation Trust Manchester UK; ^3^ Newcastle University Newcastle upon Tyne UK

## Abstract

**Objective:**

Rheumatoid arthritis (RA) is a genetically complex disease of immune dysregulation. This study sought to gain further insight into the genetic risk mechanisms of RA by conducting an expression quantitative trait locus (eQTL) analysis of confirmed genetic risk loci in CD4+ T cells and B cells from carefully phenotyped patients with early arthritis who were naive to therapeutic immunomodulation.

**Methods:**

RNA and DNA were isolated from purified B and/or CD4+ T cells obtained from the peripheral blood of 344 patients with early arthritis. Genotyping and global gene expression measurements were carried out using Illumina BeadChip microarrays. Variants in linkage disequilibrium (LD) with non‐HLA RA single‐nucleotide polymorphisms (defined as r^2^ ≥ 0.8) were analyzed, seeking evidence of *cis*‐ or *trans*‐eQTLs according to whether the associated probes were or were not within 4 Mb of these LD blocks.

**Results:**

Genes subject to *cis*‐eQTL effects that were common to both CD4+ and B lymphocytes at RA risk loci were *FADS1*,*FADS2*,*BLK*,*FCRL3*,*ORMDL3*,*PPIL3*, and *GSDMB*. In contrast, those acting on *METTL21B*,*JAZF1*,*IKZF3,* and *PADI4* were unique to CD4+ lymphocytes, with the latter candidate risk gene being identified for the first time in this cell subset. B lymphocyte–specific eQTLs for *SYNGR1* and *CD83* were also found. At the 8p23 *BLK–FAM167A* locus, adjacent genes were subject to eQTLs whose activity differed markedly between cell types; in particular, the *FAM167A* effect displayed striking B lymphocyte specificity. No *trans*‐eQTLs approached experiment‐wide significance, and linear modeling did not identify a significant influence of biologic covariates on *cis*‐eQTL effect sizes.

**Conclusion:**

These findings further refine the understanding of candidate causal genes in RA pathogenesis, thus providing an important platform from which downstream functional studies, directed toward particular cell types, may be prioritized.

Rheumatoid arthritis (RA) is a complex genetic disease in which immune tolerance becomes impaired, and an unchecked inflammatory response leads to chronic pain and damage to the synovial joints 1. Genetic variation at the *HLA–DRB1*,* HLA–DPB*, and *HLA–B* loci accounts for a large proportion of the known RA risk 2, with implications for antigen presentation to T lymphocytes 3, 4. Outside of the HLA region, accumulating data now highlight an overlap between the 101 confirmed RA risk loci and cell‐specific enhancer elements, which is maximal in CD4+ lymphocytes followed by B lymphocytes 5, 6, 7, 8. Such molecular insights support a pivotal role for both CD4+ T cell and B cell lineages in the pathogenesis of RA 9, 10, 11. Mapping cellular mechanisms of genetic risk in the disease is far from straightforward, however, because lead single‐nucleotide polymorphisms (SNPs) at associated loci are typically noncoding and intergenic, tagging linkage disequilibrium (LD) blocks that contain multiple genes 7, 12.

To prioritize causal genes, one solution is to explore associations between genetic variants and downstream molecular quantitative traits, the most proximal of which is gene expression. Thus, with respect to a putative susceptibility gene, colocalization of an expression quantitative trait locus (eQTL) with a disease risk variant implicates the gene as a candidate for disease causation 13. Data from eQTL studies in healthy human subjects have indeed informed algorithms for prioritization of candidate genes in RA 7. Importantly, however, it is now clear that the transcriptional consequences of genetic variation can manifest as cell type specificity, with potentially profound implications for disease pathogenesis 14, 15. For example, it has been observed that only 22% of *cis*‐eQTLs are consistently identified in different circulating cell subsets from healthy donors; eQTLs present in a specific cell type may not be detectable in another cell type or in whole blood—and vice versa. Moreover, a number of eQTLs can be detected only under specific conditions of cell stimulation 14, 16, 17. This suggests that the contribution of eQTL data to inferred causality among candidate genes for a given disease must increasingly be understood at a cellular level and within a relevant biologic context 18.

The suggestion that the effect size of a risk variant's influence on gene expression may depend on the environmental parameters to which cells are exposed has potentially important implications for understanding the complexities of disease induction. In RA, for example, the unmasking of eQTL effects in relevant cell populations during a transient systemic trigger might plausibly be sufficient to break immune tolerance, permitting a transition to persistent joint inflammation. Against this backdrop, we set out to reassess the biologic landscape of candidate susceptibility genes in RA by mapping *cis*‐ and *trans‐*eQTLs at 101 established RA risk loci in circulating CD4+ and B lymphocyte subsets sampled from a cohort of untreated patients with early arthritis. In so doing, we sought insight into potential common and/or cell‐specific mechanisms of genetic risk in a highly relevant biologic context, free from the confounding influences of in vivo immune modulation or ex vivo manipulation.

## Patients and Methods

**Patients.** Patients with early arthritis (all of self‐reported white ethnicity) who were attending the Newcastle Early Arthritis Cohort (NEAC) clinic in the UK were recruited into the study, and peripheral blood samples were obtained prior to the commencement of immunomodulatory therapy; individuals who were exposed to steroid treatment during the 3 months prior to recruitment or those whose ethnic origin, determined by genotype, was not of white Northern European descent were excluded from the analyses. This resulted in 71 patients being recruited between January 2008 and December 2009, and a further 273 during 2012 and 2013; the NEAC cohort has been described in detail elsewhere 19, 20, 21, 22. Initial diagnoses were validated at follow‐up visits over a median period of 20 months (range 13–25 months; duration of follow‐up >1 year in all cases), as described previously 19, 21. All patients gave their written, informed consent for inclusion into the study, which was approved by the local Regional Ethics Committee.

**Measurements of gene expression, data curation, and quality control.** Whole peripheral blood was stored at room temperature for ≤4 hours before processing. CD4+ lymphocytes were isolated from the peripheral blood by positive selection, as previously described 21, yielding a median cell purity of 98.9%. To obtain B lymphocytes, peripheral blood mononuclear cells were first isolated by density centrifugation using the Lymphoprep protocol (Axis‐Shield Diagnostics), and then subjected to positive selection using anti‐CD19 magnetic microbeads (Miltenyi Biotec). The median cell purity was 96.4%, as determined by flow cytometry (see Supplementary Figure 1, available on the *Arthritis & Rheumatology* web site at http://onlinelibrary.wiley.com/doi/10.1002/art.40393/abstract).

RNA was immediately extracted from total CD4+ T cells or B lymphocytes using an RNeasy Mini kit (prior to 2012) or AllPrep DNA/RNA Mini kit (both from Qiagen), and then subject to quality control using an Agilent 2100 Bioanalyzer (Agilent). The median RNA integrity number in the samples analyzed was 9.4. Complementary RNA generated from 250 ng total RNA (Illumina TotalPrep RNA Amplification kit) was hybridized to either an Illumina Whole Genome 6 version 3 (using CD4+ lymphocyte samples obtained prior to 2012) or a 12HT BeadChip (using CD4+ T cell samples obtained in or after 2012, and all B cell samples) (both from Illumina). The analysis was limited to probes determined to be common to both array platforms, based on unique capture sequence identifiers. Those liable to cross‐hybridization according to probe‐sequence BLAT analysis were then excluded.

Following normalization (robust spline normalization) and variance stability transformation 23, 24, batch correction of the data from CD4+ cells by linear modeling 25, and merging of the component data sets 26, principal components analysis was carried out to confirm correction for technical bias (see Supplementary Figure 2, available on the *Arthritis & Rheumatology* web site at http://onlinelibrary.wiley.com/doi/10.1002/art.40393/abstract). The raw and processed expression data used in this study are available in the Gene Expression Omnibus database (accession nos. GSE20098, GSE80513, or GSE100648; http://www.ncbi.nlm.nih.gov/geo) (a complete list of unique identifiers is provided in Supplementary Table 1, available on the *Arthritis & Rheumatology* web site at http://onlinelibrary.wiley.com/doi/10.1002/art.40393/abstract).

**Genotyping.** Genomic DNA was isolated from the peripheral blood of all patients, either from the whole blood using the Wizard genomic DNA purification kit (Promega) (for samples obtained prior to 2012) or from isolated lymphocytes in parallel with RNA extraction (AllPrep DNA/RNA Mini kit; Qiagen). Genotyping was carried out using an Illumina Human CoreExome‐24 version 1‐0 array, following the manufacturer's protocol. Samples and SNPs with a call rate of <98% were excluded. In addition, SNPs with a minor allele frequency of <0.01 or an Illumina GenomeStudio cluster separation of <0.4 were excluded from further analysis. Data were pre‐phased using SHAPEIT2 and imputed to the 1000 Genomes Phase 1, version 3, reference panel using IMPUTE2. Imputed SNPs with INFO scores of <0.8 were excluded.

**Analysis of eQTLs and covariates.** Analysis of eQTLs was limited to loci defined by the 101 lead disease‐associated variants confirmed to be present in Caucasians, as previously described by Okada et al 7. For this analysis, linear models were fitted and residual analysis was performed to verify model assumptions using the R package; Pearson's R^2^ statistics and associated *P* values were derived. Due to abundant cross‐hybridization of the expression probes and the confounding effect of copy numbers within the HLA region, we limited our analysis to non‐HLA variants. Permutation testing (10,000 permutation replicates) was carried out to derive experiment‐wide *P* values equivalent to a predetermined α value of 5%; a more relaxed (though nonetheless robust) significance threshold was also defined at an α value of 10%. This method, utilized to correct for multiple testing, proved more stringent than the standard Benjamini‐Hochberg method, which was also applied for comparison. A general linear model incorporating other potential biologic and clinical parameters, including age, sex, C‐reactive protein (CRP) level, and swollen joint count, permitted evaluation of the robustness of the eQTLs in relation to inflammation markers and other potential covariates.

**Comparisons with published data sets.** Published eQTL data sets were identified using PubMed literature searches. Results were cross‐checked and validated with reference to the GTEx Portal database (available at http://gtexportal.org) 27.

## Results

**Mapping of eQTLs at RA risk loci in lymphocytes of treatment‐naive patients with early arthritis.** Expression data from primary peripheral blood lymphocytes were available for a total of 344 genotyped patients with early arthritis; available data on CD4+ lymphocytes were limited to 249 of the patients, data on B lymphocytes were available for 242 of the patients, and paired data were available for 147 of the patients. The baseline clinical characteristics and diagnoses of all patients are summarized in Table 1. After quality control procedures were applied, a total of 1,227 genotyped variants in LD (defined as r^2^ ≥ 0.8) with lead RA‐associated SNPs were considered. Filtered expression probes whose start sites mapped to within 4 Mb of LD blocks (as defined in Patients and Methods) were initially measured to identify *cis*‐acting eQTLs. In a secondary analysis of *trans*‐eQTLs, those with start sites >4 Mb from the same LD blocks were evaluated in a similar manner.

**Table 1:** Characteristics of the patients with early arthritis^a^

	RA (n = 124)	Non‐RA inflammatory arthritis (n = 113)	Noninflammatory arthritis (n = 107)	*P* b
Age, years	59 (48–73)	51 (39–63)	52 (44–57)	<0.001
Sex, % female	69	61	81	<0.001
Duration of symptoms, weeks	12 (8–27)	12 (6–25)	24 (8 to >52)	0.03
CRP, gm/liter	11 (5–26)	8 (5–19)	<5 (<5–8)	<0.001
ESR, mm/minute	26 (12–49)	19 (7–34)	8 (4–20)	<0.001
TJC	6 (3–12)	2 (1–6)	3 (0–8)	0.001
SJC	2 (1–6)	1 (0–3)	0 (0–0)	<0.001
DAS28	4.68 (3.5–5.5)	NA	NA	NA
RF positive, %	57	7	14	<0.001
ACPA positive, %	48	2	1	<0.001
Non‐RA diagnosis, %				
Osteoarthritis	–	–	55	–
Other, noninflammatory arthritis	–	–	45	–
Spondyloarthropathy (PsA, AS, EA)	–	68	–	–
Crystal arthropathy	–	9	–	–
Other, inflammatory arthritis	–	20	–	–
Undifferentiated arthritis	–	3	–	–

a Patients with early arthritis are stratified by diagnostic category, including non–rheumatoid arthritis (RA) subclassifications. Except where indicated otherwise, values are the median (interquartile range). CRP = C‐reactive protein; ESR = erythrocyte sedimentation rate; TJC = tender joint count (of 28 joints); SJC = swollen joint count (of 28 joints); DAS28 = Disease Activity Score in 28 joints; NA = not applicable; RF = rheumatoid factor; ACPA = anti–citrullinated peptide autoantibody; PsA = psoriatic arthritis; AS = ankylosing spondylitis; EA = enteropathic arthritis.

b *P* values were based on Kruskal‐Wallis nonparametric analysis of variance for continuous variables, and chi‐square test for dichotomous variables.

**Figure 1:**
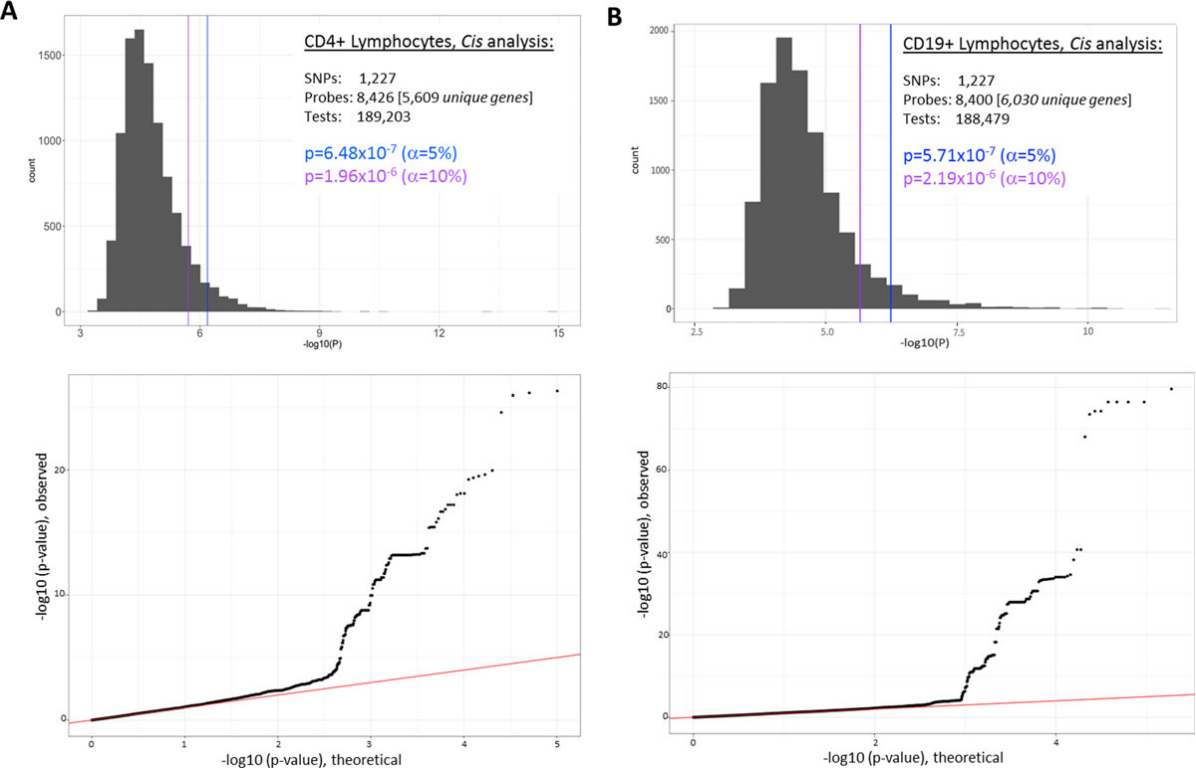
Determination of experiment‐wide significance of *cis*‐acting expression quantitative trait loci (*cis*‐eQTLs) in CD4+ T lymphocytes **(A)** and CD19+ B lymphocytes **(B)**. Top, Histograms summarize the data from 10,000 permutation replicates, each derived from the indicated number of single‐nucleotide polymorphisms (SNPs) and expression probes, and the final number of included tests. *P* values at the α = 5% and α = 10% thresholds are shown. Bottom, QQ plots depict expected *P* value distributions under the null hypothesis (red line) versus observed distributions. Analogous plots for analyses of *trans*‐eQTLs are shown in Supplementary Figure 3 (available on the *Arthritis & Rheumatology* web site at http://onlinelibrary.wiley.com/doi/10.1002/art.40393/abstract).

Permutation testing was carried out using 10,000 permutation replicates for each analysis in each lymphocyte subset. This allowed us to account for multiple testing, in which the total number of tests for each cell type corresponded to the number of unique SNP–gene pairs in the analyses of *cis*‐ or *trans*‐acting eQTLs across the prespecified loci, after data processing and quality control had been performed. The maximum value of the test statistic (minimum nominal *P* value) across the total number of tests in each permutation replicate was recorded, and significance thresholds exceeding 5% or 10% in each permutation replicate were determined. This procedure resulted in experiment‐wide *P* value thresholds (α = 5% or α = 10%) that were used to define evidence of eQTLs in each cell type, as summarized in Figure 1 (for *cis*‐eQTL analyses) and in Supplementary Figure 3 (for *trans*‐eQTL analyses; available on the *Arthritis & Rheumatology* web site at http://onlinelibrary.wiley.com/doi/10.1002/art.40393/abstract).

In total, 213 *cis*‐acting significant SNP–transcript associations were identified in CD4+ lymphocytes (α = 5%), corresponding to 10 unique genes at 7 established RA risk loci; 194 *cis*‐eQTLs were similarly identified in B lymphocytes (α = 5%), also corresponding to 10 unique genes at 7 loci. The *cis*‐eQTL effects for *FADS1*,* FADS2*,* FCRL3*,* BLK*,* ORMDL3*,* GSDMB*, and *PPIL3* were robust in both CD4+ and B lymphocytes at RA risk loci. The eQTLs acting on 3 genes (*METTL21B*,* IKZF3,* and *JAZF1*) were unique to CD4+ T lymphocytes in this population, with *PADI4* also subject to a convincing effect exclusively in this cell type despite falling marginally short of the α = 10% threshold by permutation analysis; the latter gene encodes a peptidylarginine deiminase enzyme, and therefore is of interest in the pathogenesis of RA 28.

**Figure 2:**
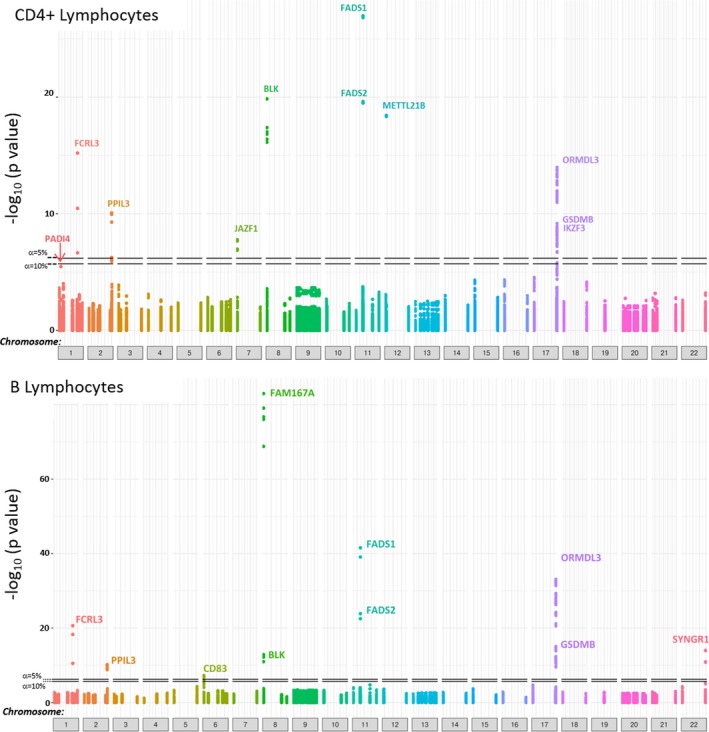
Manhattan plots depict the 101 rheumatoid arthritis risk loci analyzed and *P* values for the significance of single‐nucleotide polymorphism (SNP)–probe pairs (denoted by different‐colored dots) among CD4+ T lymphocytes (top) and B lymphocytes (bottom) in patients with early arthritis. Human Genome Organisation gene symbols for SNP–probe pairs, or groups thereof, that approached or reached experiment‐wide significance (at thresholds of α = 5% and α = 10% [horizontal lines]) are indicated, permitting comparison of expression quantitative trait loci between cell types.

At the 8p23 locus, *FAM167A* was, in contrast to the neighboring *BLK* gene, shown to be subject to *cis* regulation only in B lymphocytes, and *SYNGR1* and *CD83* eQTLs were also specific to this cell type. These data are summarized in Tables 2 and 3 and depicted as Manhattan plots in Figure 2. No *trans*‐eQTLs achieved experiment‐wide significance thresholds, either in CD4+ T lymphocytes or in B lymphocytes.

**Table 2:** Summary of CD4+ T lymphocyte *cis*‐eQTL genes^a^

Gene	Lead eQTL SNP	Locus	Minor allele (MAF)	*P* b	R^2^ in relation to RA index SNP	Total no. of significant SNPs for probe^c^
*FADS1*	rs968567	11q12	T (0.177)	1.06 × 10^–27^	1.0	3
*BLK*	rs922483	8p23	T (0.426)	1.41 × 10^–20^	0.805	9
*FADS2*	rs968567	11q12	T (0.177)	2.40 × 10^–20^	1.0	3
*METTL21B*	rs701006	12q13–q14	A (0.408)	3.65 × 10^–19^	1.0	3
*FCRL3*	rs2210913	1q23	C (0.457)	6.24 × 10^–16^	0.873	12
*ORMDL3*	rs4795397	17q12–q21	G (0.482)	1.07 × 10^–14^	0.959	66
*PPIL3*	rs6757776	2q33	G (0.103)	8.60 × 10^–11^	1.0	16
*GSDMB*	rs4795397	17q12–q21	G (0.314)	6.92 × 10^–10^	0.969	88
*IKZF3*	rs1453559	17q12–q21	C (0.453)	2.52 × 10^–9^	0.801	23
*JAZF1*	rs4722758	7p15	G (0.195)	1.70 × 10^–8^	1.0	15
*PADI4*	rs2240339	1p36	T (0.418)	3.37 × 10^–6^ d	0.923	*–*

a Microarray probe targets are shown as Human Genome Organisation gene symbols. Lead expression quantitative trait locus (eQTL) single‐nucleotide polymorphisms (SNPs) and loci are also shown, along with their minor allele and minor allele frequency (MAF). Rheumatoid arthritis (RA) index SNPs were those listed in the report by Okada et al (see ref. 7).

b The permuted significance thresholds of α = 5% and α = 10% equate to *P* = 6.48 × 10^−7^ and *P* = 1.96 × 10^–6^, respectively (see Figure 1).

c Based on a threshold of α = 10%.

d Data for the PADI4 eQTL fell marginally short of the α = 10% threshold.

**Table 3:** Summary of CD19+ B lymphocyte *cis*‐eQTL genes^a^

Gene	Lead eQTL SNP	Locus	Minor allele (MAF)	*P* b	R^2^ with RA index SNP	Total no. of significant SNPs for probe^c^
*FAM167A*	rs4840568	8p23	A (0.264)	2.48 × 10^–80^	0.817	9
*FADS1*	rs968567	11q12	T (0.177)	1.96 × 10^–41^	1.0	3
*ORMDL3*	rs9906951	17q12–q21	C (0.383)	2.29 × 10^–35^	0.881	66
*FADS2*	rs968567	11q12	T (0.177)	6.60 × 10^–25^	1.0	3
*FCRL3*	rs2210913	1q23	T (0.482)	3.09 × 10^–22^	0.839	12
*GSDMB*	rs12936231	17q12–q21	G (0.434)	7.21 × 10^–16^	0.862	66
*SYNGR1*	rs909685	22q13	A (0.31)	3.66 × 10^–14^	1.0	5
*BLK*	rs2618476	8p23	C (0.25)	3.02 × 10^–13^	0.958	9
*PPIL3*	rs2141331	2q33	T (0.097)	1.13 × 10^–10^	0.943	8
*CD83*	rs78242827	6p23	C (0.058)	2.72 × 10^–8^	1.0	20

a Microarray probe targets are shown as Human Genome Organisation gene symbols. Lead expression quantitative trait locus (eQTL) single‐nucleotide polymorphisms (SNPs) and loci are also shown, along with their minor allele and minor allele frequency (MAF). Rheumatoid arthritis (RA) index SNPs were those listed in the report by Okada et al (see ref. 7).

b The permuted significance thresholds of α = 5% and α = 10% equate to *P* = 5.71 × 10^−7^ and 2.19 × 10^−6^, respectively (see Figure 1).

c Based on a threshold of α = 10%.

**Figure 3:**
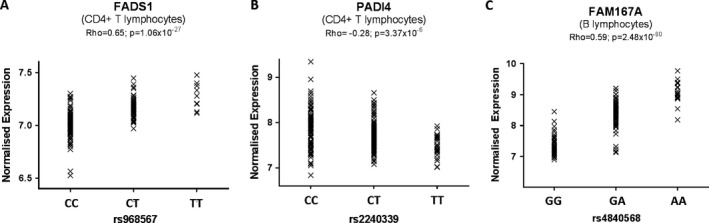
Representative examples of expression quantitative trait loci (eQTLs). Plots of normalized individual gene expression, along with their Spearman's rho statistics and *P* values for association, are shown for the lead eQTL single‐nucleotide polymorphisms acting on *FADS1* **(A)** and *PADI4* **(B)** in CD4+ T lymphocytes and *FAM167A* **(C)** in B lymphocytes.

Representative examples of eQTL plots are depicted in Figure 3, and a comprehensive list of all SNP–probe associations that remained significant after Benjamini‐Hochberg correction for multiple testing is provided in Supplementary Tables 2 and 3 (available on the *Arthritis & Rheumatology* web site at http://onlinelibrary.wiley.com/doi/10.1002/art.40393/abstract), in which significance thresholds of α = 5% and α = 10% by permutation testing are also indicated. Supplementary Table 4 (http://onlinelibrary.wiley.com/doi/10.1002/art.40393/abstract) summarizes this information, listing all significant eQTL SNPs (and associated genes) in relation to the index SNPs reported by Okada et al 7. Limiting any or all of the above analyses to samples for which paired CD4+ and B lymphocytes were available (n = 147) had no substantial effect on the eQTL genes identified, although some associations ceased to reach experiment‐wide significance due to diminished power (see Supplementary Tables 5 and 6, http://onlinelibrary.wiley.com/doi/10.1002/art.40393/abstract).

**Comparison of eQTLs with published data sets.** Our findings were considered in light of a number of human eQTL studies for which significant SNP–probe combinations are in the public domain. These included analyses of *cis*‐eQTLs in primary CD4+ and B lymphocytes. Murphy et al studied genome‐wide expression in positively selected whole CD4+ lymphocytes from 200 non‐Hispanic white subjects, comprising young adults with asthma and their first‐degree relatives 29. Hu et al examined paired expression limited to 270 genes in resting CD4+ lymphocytes and CD3/CD28‐stimulated effector memory CD4+ lymphocytes from healthy donors; genes were selected based on their proximity to 157 SNPs with known autoimmune disease associations (including with RA) 30. Raj et al reported genome‐wide eQTL data in positively selected CD45RO− (naive) CD4+ lymphocytes from 200 healthy European Americans 31, and a similar analysis, by Kasela et al, was conducted in whole CD4+ (and CD8+) T cells 32. Another study, by Fairfax et al 14, demonstrated the presence of eQTLs in primary B cells from 288 healthy Europeans. Studies by Dixon and colleagues 33, 34 presented cumulative data from Epstein‐Barr virus–transformed human B cells (lymphoblastoid cell lines), and a large meta‐analysis was conducted to compare studies performed in the whole blood of predominantly healthy volunteers 35. Finally, our data were considered in the context of the GTEx resource database 27.

Overlap between the genes subject to *cis*‐eQTLs in these studies compared with those identified in our own study is illustrated in Supplementary Figure 4 (available on the *Arthritis & Rheumatology* web site at http://onlinelibrary.wiley.com/doi/10.1002/art.40393/abstract). Reassuringly, all of the *cis‐*eQTL genes identified in patients with untreated early arthritis replicated the findings reported in at least one of the comparator studies. Strong independent validation of CD4+ lymphocyte–specific associations was provided with regard to 9 of the genes. Among these, RA risk loci at 11q12 and at 17q12–21 were each observed to harbor pairs of apparently coregulated genes, *FADS1*/*FADS2* and *ORMDL3*/*GSDMB*, respectively. Moreover, at the 17q12–21 locus, *IKZF3* was confirmed to be subject to a highly significant eQTL effect in CD4+ lymphocytes 32. When a more lenient (but nonetheless robust) method for multiple test correction was employed, we highlighted, for the first time, an association between *PADI4* expression and genotype at the 1p36 locus specifically in CD4+ T cells, its having previously been identified only in whole blood. Our findings with respect to *FAM167A*,* SYNGR1*, and *CD83* corroborate those in the only other study of primary human B lymphocytes, by Fairfax et al 14, and although the same eQTLs have been noted in mixed cell populations of whole blood 35, no study (including our own) has yet replicated them in CD4+ T lymphocytes.

**Lack of significant impact of clinical covariates on eQTLs.** Because differential eQTL effect sizes have been observed in paired CD4+ T cells from healthy donors according to whether T cell receptor–mediated stimulation of the cells was undertaken ex vivo prior to RNA extraction 30, we hypothesized that certain clinical covariates, and/or the activation status of circulating CD4+ T cells, might have a similar influence in vivo. The clinical parameters considered included age, sex, CRP level, and erythrocyte sedimentation rate (as indicators of the systemic acute‐phase response), as well as disease phenotype (RA versus non‐RA). In the patient subgroup for whom CD4+ lymphocyte expression data were available, normalized transcript levels of CD25, CD69, and interferon‐γ, as measured by microarray, were also considered as surrogates of the CD4+ T cell activation status. The incorporation of each of these covariates, in turn, into linear models made no difference to the final eQTL list (as shown in Tables 2 and 3), and individual regression slopes were robust to their inclusion (representative examples are depicted in Supplementary Figure 5, available on the *Arthritis & Rheumatology* web site at http://onlinelibrary.wiley.com/doi/10.1002/art.40393/abstract). Consistent with these findings, lists of genes subject to *cis*‐eQTL effects did not vary substantially when patients with RA and those with alternative diagnoses were considered independently (results not shown). Thus, eQTLs were robust to clinical and biologic covariates in our study, and no evidence of early disease–specific eQTLs at RA risk loci was found.

## Discussion

We present the first eQTL analysis of primary lymphocytes from donors presenting with untreated, suspected inflammatory arthritis—a context highly relevant for the purpose of unravelling genetic risk mechanisms in RA. Several important observations can be made on the basis of our findings.

CD4+ and B lymphocytes in this setting exhibit distinct but overlapping eQTLs at confirmed RA risk loci (Tables 2 and 3). The specificity of an eQTL effect for one cell type may simply be a reflection of the lack of expression of a gene by a comparator cell, but probe‐level microarray data suggest that reported genes were expressed in both CD4+ and B lymphocytes in our study. Therefore, the cell‐specific effects that we observed for *METTL21B*,* IKZF3*,* JAZF1*, and *PADI4* (in CD4+ lymphocytes) and *FAM167A*,* SYNGR1*, and *CD83* (in B lymphocytes) may indicate differential regulatory functions of disease risk variants between lineages.

Strikingly, at the common *BLK–FAM167A* autoimmune locus at 8p23, we found that 2 adjacent genes were subject to eQTLs whose activity differed between cell types: the *FAM167A* effect displayed robust B lymphocyte specificity and was absent in CD4+ lymphocytes, whereas the *BLK* effect that was prominent in CD4+ T lymphocytes was less prominent among B lymphocytes (compare Table 2 and Table 3, and see Figure 2). The most strongly associated SNPs differed between cell types at this locus—a finding that was maintained among patients for whom paired cell–specific data were available (as in Supplementary Tables 5 and 6, http://onlinelibrary.wiley.com/doi/10.1002/art.40393/abstract), potentially signifying the presence of mechanistically distinct regulatory variants in strong LD. Nonetheless, the results of our study also contribute to an emerging picture in which eQTLs can regulate the expression of more than one gene at disease‐associated loci, examples being found at 11q12 and 17q12–21. This is consistent with the concept that key genetic variants may act as “master regulators” of gene expression.

Our findings provide an important platform from which downstream functional studies may be directed toward particular cell types. For example, elucidating the relevance of the *METTL21B* gene product in CD4+ T cell function would now seem a priority, given our findings confirming a pronounced eQTL effect on this gene in this cell type. Alternative causal candidate genes known, to date, to be favored at the 12q13–q14 locus are *CDK4* and *CYP27B1*, based on their respective functions in cell‐cycle progression and vitamin D metabolism 7; however, since neither of these genes were shown to be subject to prominent eQTL effects, despite the growing body of literature discussing their functions, it seems justified to consider *METTL21B* as an alternative candidate gene in CD4+ T cells.

A similar case for both *CD83* and *SYNGR1* in B lymphocytes might also be made. *CD83* encodes a transmembrane member of the immunoglobulin superfamily expressed widely on dendritic cells, but also on activated lymphocytes; its important role in regulating B lymphocyte development and effector function is only now beginning to be understood 36. *SYNGR1* is an integral membrane protein associated with presynaptic vesicles in neuronal cells, and its function in lymphoid cells remains obscure. However, caution should be exercised when interpreting transcript eQTLs in isolation 37, and validation of our findings at the protein level should be prioritized. This was amply illustrated by Simpfendorfer et al, who, similar to our findings at the 8p23 locus, highlighted *BLK* transcript expression as subject to an eQTL in lymphocytes; however, the CD4+ T cell–specific effect was not sustained at the protein level in these cells. By measuring allelic expression imbalance, those authors went on to demonstrate a robust eQTL for both RNA and protein expression in naive/transitional B cell subsets isolated from umbilical cord blood, which was less evident in whole B cells, suggesting that disease risk is conferred during early B cell development rather than by CD4+ T cells 38, potentially via dysregulated B cell receptor signaling 39.

Our study is the first to provide evidence of an eQTL SNP in CD4+ lymphocytes that was in perfect LD with the RA‐associated variant at the 1p36 locus, a variant that regulates *PADI4* gene expression. The *PADI4* gene has already been recognized as a strong causal candidate for the disease, encoding peptidylarginine deiminase 4, a key enzyme involved in posttranslational citrullination of arginine residues that yields neoepitopes against which RA‐specific anti–citrullinated peptide autoantibodies may be raised 28. However, distinct mechanisms of CD4+ lymphocyte dysregulation now warrant further investigation 40.

Similarly, the finding that *IKZF3* is subject to an eQTL in CD4+ lymphocytes is, to our knowledge, a novel observation and is intriguing, given the proven role of the transcription factor product of this gene in regulating interleukin‐10 production by these cells 41.

Conceiveably, our observations with regard to *PADI4* and *IKZF3* could be interpreted as evidence that putatively common causal SNPs augment gene expression in CD4+ T cells uniquely under the particular biologic and/or environmental circumstances of early arthritis. However, our analysis of interactions between specific biologic covariates and eQTL effects did not support such an interpretation: in particular, the *IKZF* rs9916765 eQTL slope gradient was unaffected by markers of systemic inflammation, T cell activation, or clinical diagnosis (see Supplementary Figures 5D–F, http://onlinelibrary.wiley.com/doi/10.1002/art.40393/abstract). While this could be seen as surprising, given the previously reported differences between CD4+ T cell eQTLs according to activation status in vitro 30, the contrastingly cross‐sectional nature of our study, which focused on unstimulated ex vivo cells from systemically inflamed and uninflamed peripheral blood samples, render the findings complimentary rather than contradictory, in our view. Indeed, the fact that eQTL effects did not differ according to disease classification (e.g., RA versus non‐RA) in our early arthritis population recalls the findings in a study by Peters et al, whereby inflammatory bowel disease–specific eQTLs resided outside of known risk loci for that condition 42. Further work is therefore needed to elucidate the mechanisms by which eQTL effects may wax or wane at a cellular level within the in vivo environment.

Our data extend the understanding of the causal candidate gene landscape in early RA, highlighting several such candidates that now deserve further investigation in defined primary lymphocyte populations. In the future, the possibility that eQTL effects may exhibit heterogeneity between subsets of CD4+ T and/or B lymphocytes should be considered, since these populations are well‐known to comprise functionally diverse compartments. Moreover, it is likely that larger integrative studies, including meta‐analyses of accumulating lymphocyte eQTL data sets in relevant populations, will be required to expand on this. Such work will have additional value in the identification of *trans*‐eQTL effects, which, because of power considerations, we were not able to address in the present study.

## Author Contributions

All authors were involved in drafting the article or revising it critically for important intellectual content, and all authors approved the final version to be published. Dr. Pratt had full access to all of the data in the study and takes responsibility for the integrity of the data and the accuracy of the data analysis.

### Study conception and design

Thalayasingam, Isaacs, Barton, Pratt.

### Acquisition of data

Thalayasingam, Nair, Massey, Anderson, Diboll, Pratt.

### Analysis and interpretation of data

Thalayasingam, Nair, Skelton, Massey, Clark, Lendrem, Reynard, Cordell, Eyre, Pratt.
