# Supplementary material for: CD4+ and B Lymphocyte Expression Quantitative Traits at Rheumatoid Arthritis Risk Loci in Patients With Untreated Early Arthritis: Implications for Causal Gene Identification
Source: Arthritis Rheumatol. 2018 Jan 30;70(3):361–70. doi: 10.1002/art.40393 (PMC5888199; doi:10.1002/art.40393)
Supplement: Supplementary file 1 [file ART-70-361-s001.docx]

**CD4+ and B lymphocyte expression quantitative traits at rheumatoid arthritis risk loci in untreated early arthritis: implications for causal gene identification?**

Thalayasingam N. et al

**Supplementary Figures.**

**
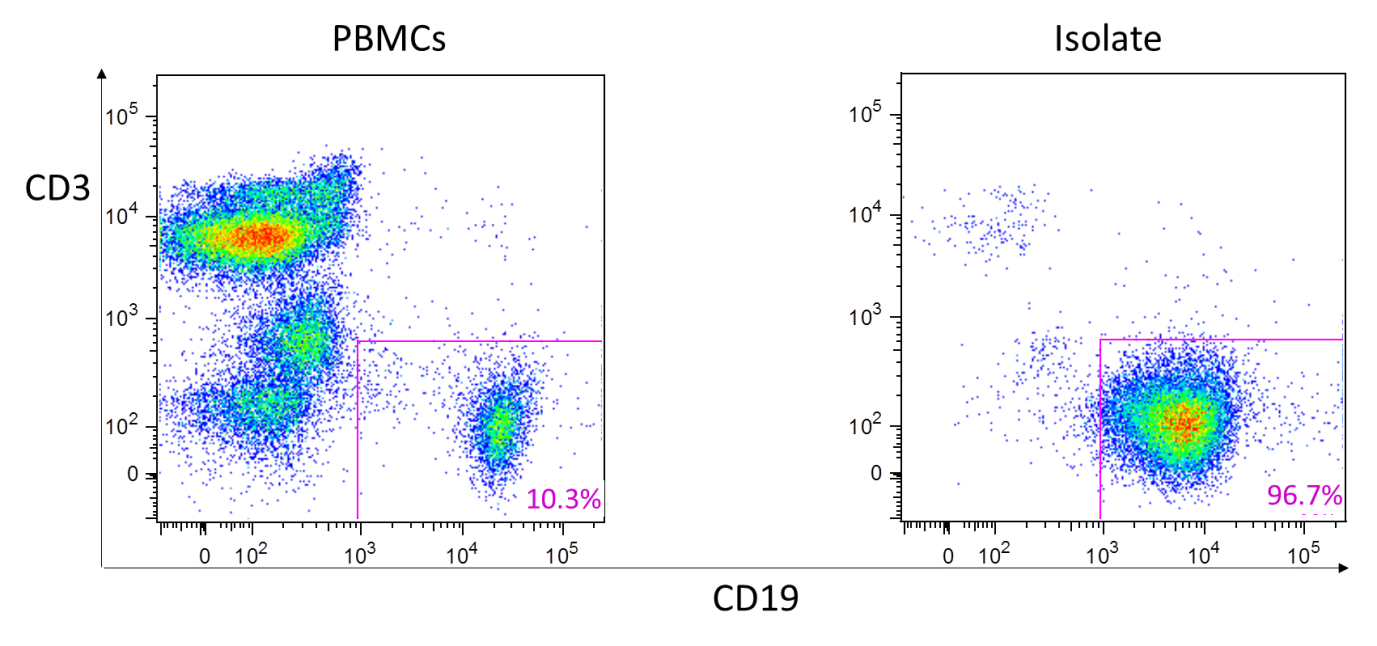
**

***Supplementary Figure 1.*** *FACS plots illustrating B lymphocyte purity in fresh cellular isolates. Percentage purity of CD19+ CD3- B lymphocytes within overall population is indicated in lower right gate of each panel. A. PBMCs of freshly isolated peripheral blood mononuclear cells (PBMCs). B. PBMCs following anti-CD19 magnetic bead positive selection (96.7% purity), from which RNA was subsequently extracted. Median B lymphocyte purity for this study was 94.9% in this study.*


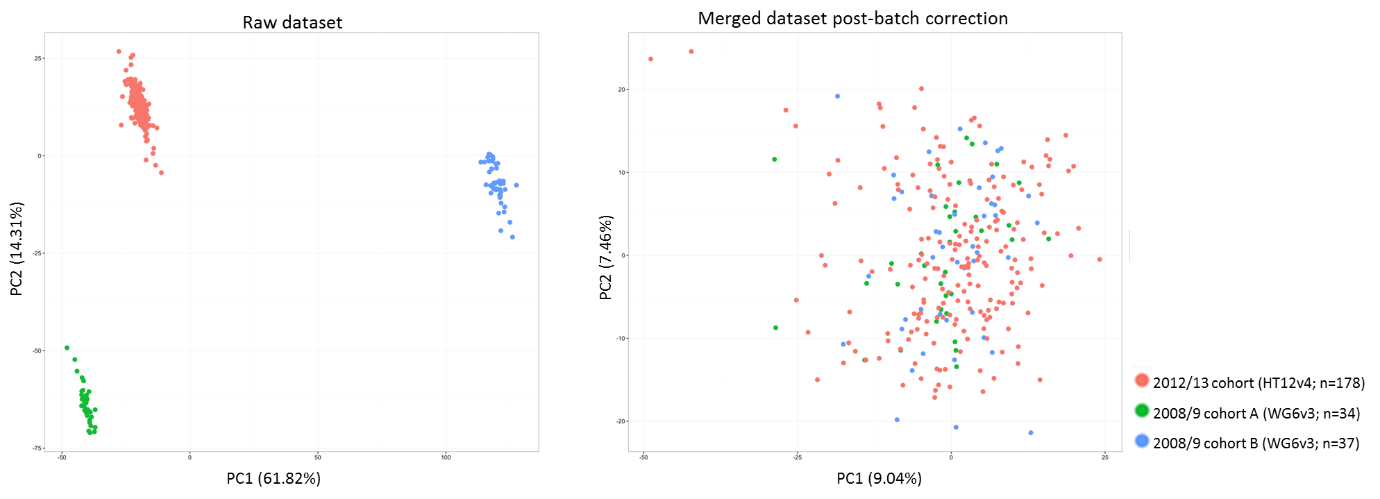


***Supplementary Figure 2****.* *Principal component analysis of CD4+ T cell microarray gene expression data for 247 individuals labelled according to processing batch (see Methods). The final early arthritis cohort analysed comprised 71 patients recruited in 2008-9 and processed in 2 separate batches using the Illumina WG6v3 BeadChip array (see reference 30; blue and green coordinates), and 176 newly-recruited patients processed in a single batch using the Illumina HT12v4 BeadChip array (red coordinates). Compared with raw, uncorrected data (left panel), batch-correction as described removes variability due to technical bias (right panel).B cell gene expression data was processed in a single batch (not shown).*

***
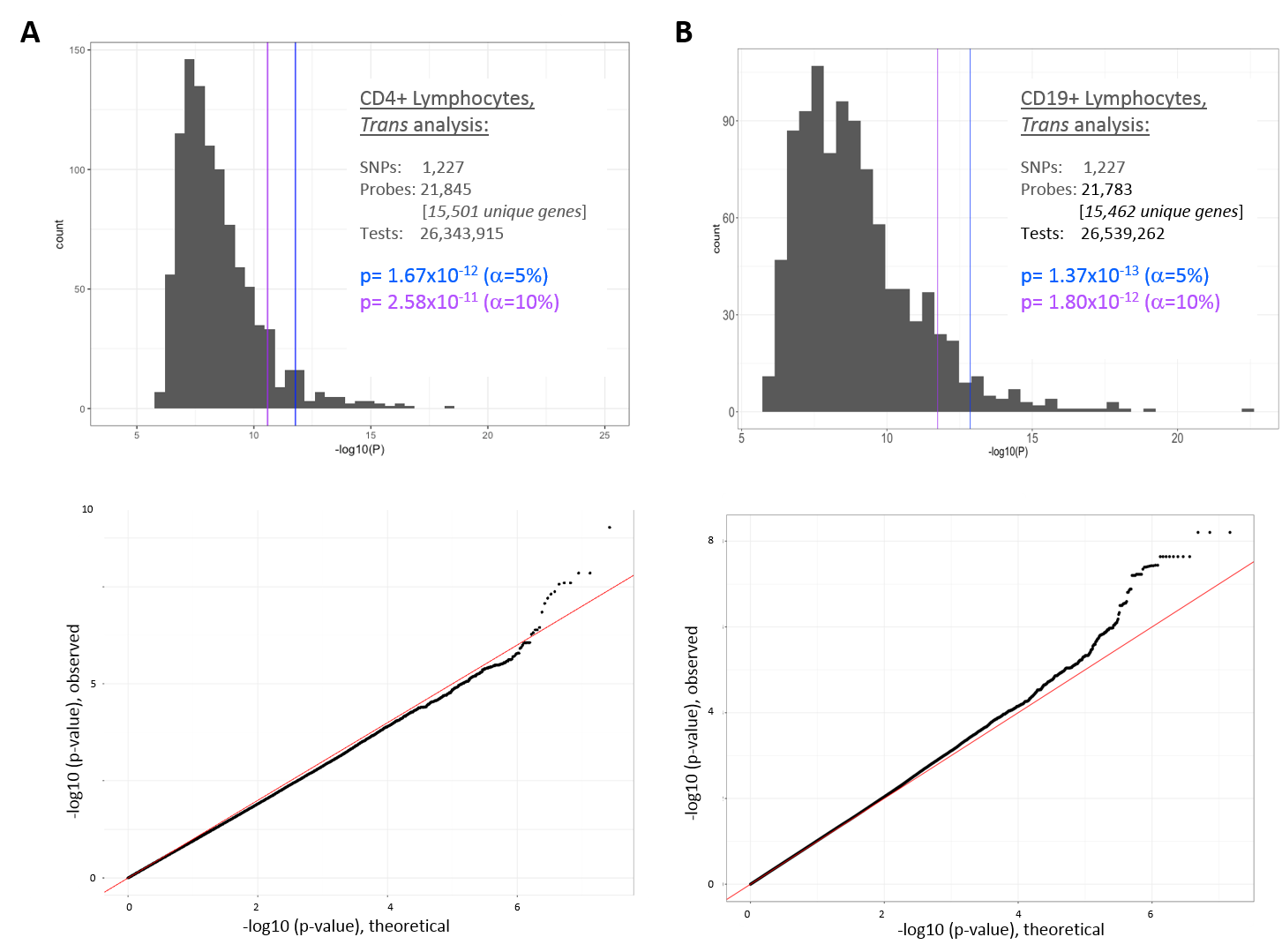
Supplementary Figure 3****. Determination of experiment-wide p-values used for trans eQTL analysis in CD4+ T lymphocytes (****A****) and B lymphocytes (****B****). Histograms in upper panels summarise 10,000 permutation replicates, each derived from indicated number of SNPs/expression probes and final number of included tests; p-values indicative of α = 5% and 10% are shown. QQ plots in lower panels depict expected p value distributions under the null hypothesis (red lines) versus observed distributions.*

***
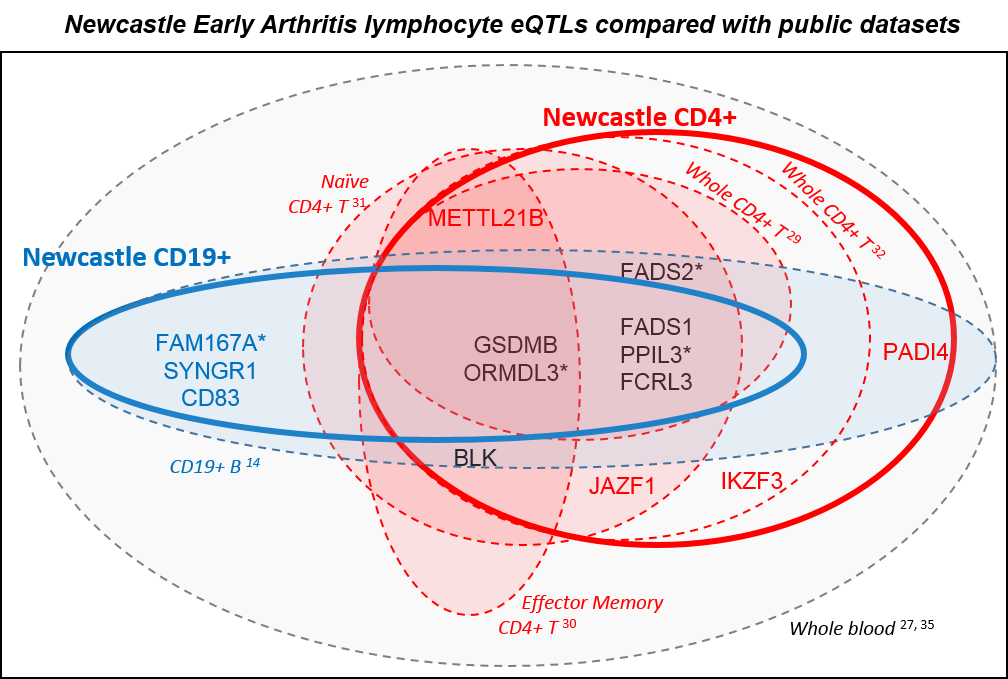
Supplementary Figure 4****. Venn diagram illustrating overlap of cis eQTLs discovered amongst early arthritis patients in the current study (solid-lined ellipses) in relation to indicated published peripheral blood eQTL datasets (dotted-lined ellipses, as referenced) from CD4+ T lymphocytes (red), B lymphocytes (blue) and whole blood (grey). Text colour of indicated genes denotes whether subject to eQTL in both cell types (black), uniquely in CD4+ T lymphocytes (red) or uniquely in B lymphocytes (blue). *Asterisked genes were previously identified in lymphoblastoid cell lines (refs 33, 34). See main document for references.*


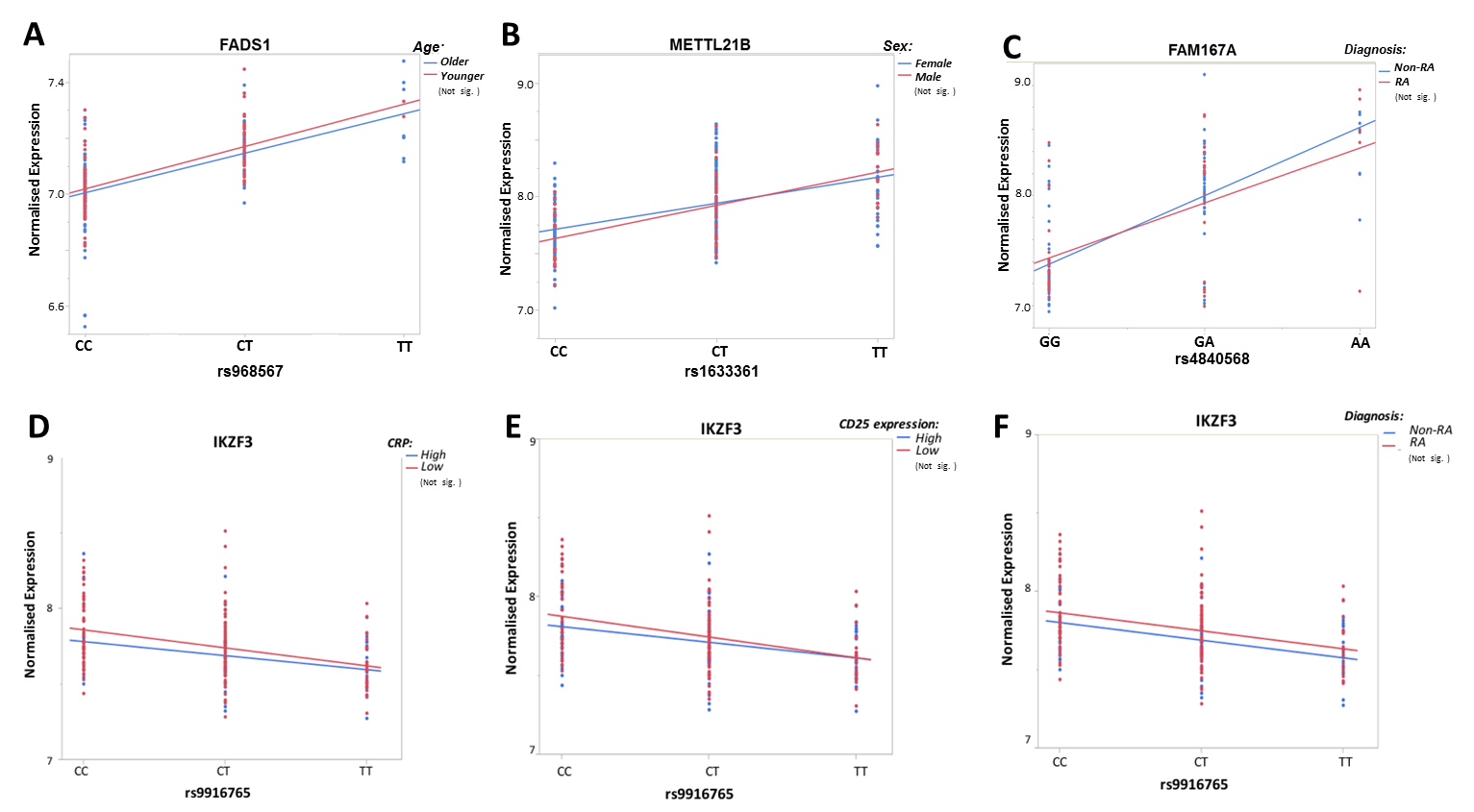


***Supplementary Figure 5****. For each gene’s lead SNP-probe pair, eQTL effects were not found to be significantly impacted by the inclusion of clinical covariates (age, sex, CRP), markers of CD4+ lymphocyte activation (normalised CD25, CD69 and IFNg expression) and diagnosis (RA versus non-RA). All above-listed covariates were considered for each SNP-probe pair reported in Tables 2 & 3. Exemplar plots for (****A****) FADS1, CD4+ lymphocytes; (****B****) METTL21, CD4+ lymphocytes; (****C****) FAM167A, B lymphocytes; and (****D-F****) IKZF3, CD4+ lymphocytes, depict regression lines adjusted for named co-variate (indicated red and blue comparators, with continuous variables dichotomised around median value for presentation purposes). No significant differences between regression slopes were detected in any of the analyses (analysis of co-variance).*
