# Supplementary material for: CD4+ and B Lymphocyte Expression Quantitative Traits at Rheumatoid Arthritis Risk Loci in Patients With Untreated Early Arthritis: Implications for Causal Gene Identification
Source: Arthritis Rheumatol. 2018 Jan 30;70(3):361–70. doi: 10.1002/art.40393 (PMC5888199; doi:10.1002/art.40393)
Supplement: Supplementary file 2 [file ART-70-361-s002.docx]

**CD4+ and B lymphocyte expression quantitative traits at rheumatoid arthritis risk loci in untreated early arthritis: implications for causal gene identification?**

Thalayasingam N. et al

**Supplementary Tables**

**Supplementary Table 1.**

*Raw data was extracted from previously published microarray (where paired genotype data available; GSE20098) and newly performed microarray (GSE80513, GSE100648). Table lists unique sample IDs for participants and source dataset GEO accession numbers.*

**Supplementary Table 2.**

*Significant cis SNP-probe associations for all study participants amongst whom CD4+ T lymphocyte eQTL analysis was performed (complete CD4+ T Lymphocyte cohort; n=249).*

*rs numbers for eQTL SNPs and index RA SNP (Okada et al Nature 2014; reference 7 in main article) are provided, along with Illumina probe ID and HUGO gene symbol. Raw p value for the association is given each case, along with Benjamini-Hochberg-corrected p value. α=5% and α=10% thresholds by permutation analysis (6.48x10^7^ and 1.98x10^-6^) indicated. SNP-Probe pairs ranked by p value.*

**Supplementary Table3.**

*Significant cis SNP-probe associations for all study participants amongst whom B lymphocyte eQTL analysis was performed ("Complete B Lymphocyte cohort;" n=242). Annotation as for Supplementary Table 2.*

*a=5% and a=10% thresholds by permutation analysis (2.11x10^-7^ and 1.07x10^-6^) indicated. SNP-Probe pairs ranked by p value.*

**Supplementary Table 4.** Summary of significant eQTL SNPs and associated genes, listed in relation to index RA-associated SNPs (with which they are in linkage disequilibrium) reported by Okada *et al* (ref. 7).

Tables 4[i] and 4[ii] depict data for CD4+ T cells and B cells, respectively.

***Table 4[i].***

**
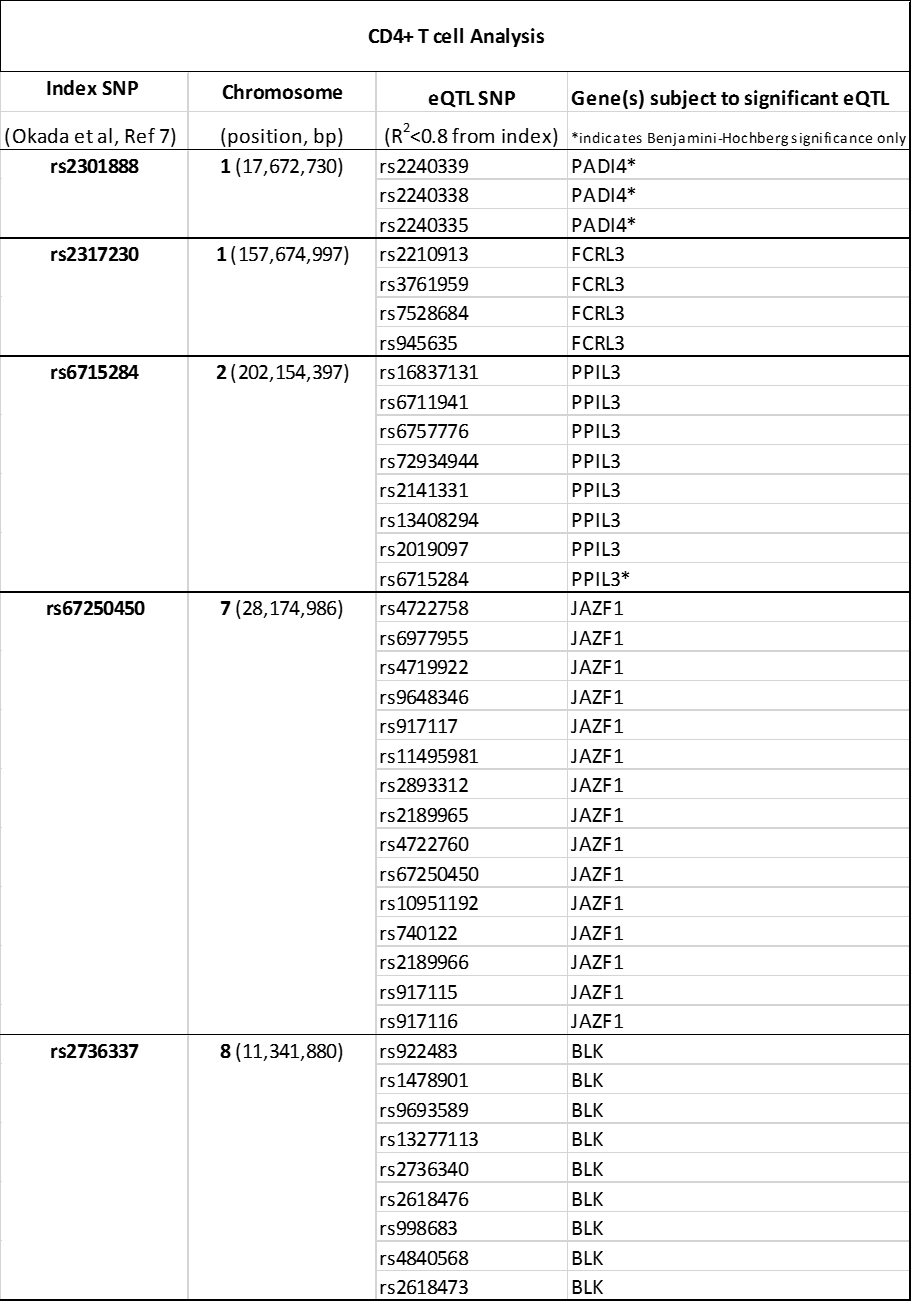
**

***Table 4[ii]***


 **Supplementary Table 5.** *Significant cis SNP-probe associations (α=10% by permutation analysis) in CD4+ T lymphocytes for 147 study participants amongst whom paired CD4+ T and B-lymphocyte expression data were available. Annotation as for Table 2.*

**Supplementary Table 6.**

*Significant cis SNP-probe associations (a=10% by permutation analysis) in CD19+ B lymphocytes for 147 study participants amongst whom paired CD4+ T and B-lymphocyte expression data were available. Annotation as for Table 2.*
